# Case of Peritonitis with Probable Intestinal Perforation

**Published:** 1853-06

**Authors:** 


*Case of Peritonitis with probable Intestinal Perforation.*—Dr. Wood wished to call the attention of the college to a case of peritonitis which had recently been treated, under his supervision, in the Pennsylvania Hospital. But before proceeding, he would remind the Fellows that, on a previous occasion, he had offered them a brief notice of a case of the same disease, occurring suddenly during apparent convalescence from typhoid or enteric fever, and terminating favorably under the opiate treatment recommended by Drs. Stokes and Graves of Dublin, together with a large blister. This notice may be seen in the *Summary of the Transactions of the College* for Nov. 5, 1850 (Vol. I.,N. S., page 32). As is well known to many of the Fellows, the patient was a highly respectable young medical gentleman; and of the nature of the case there was little room to doubt. The one now to be noticed is of a less certain character, but will be found to present features which render it highly probable that the Inflammation of the peritoneum resulted from perforation of the bowels. For the dates in the case, Dr. Wood was indebted to his young friend, Dr. Penrose, one of the resident physicians of the Hospital, who had the immediate charge of the patient.

At the commencement of my term of attendance in the hospital, observed Dr. Wood, the patient had been several days in the house, laboring under a slight fever, which had been preceded before his admission by a feeling of general uneasiness, weakness, and loss of appetite. There had been no epistaxis or diarrhœa. When I first saw him, his pulse was somewhat accelerated, his skin warmer than in health, the tongue lightly furred, and the abdomen moderately meteoric, with slight pain on pressure. The symptoms were those of a mild case of enteric fever at the close of the first or commencement of the second week; but there were no sudamina, no red spots on the abdomen or elsewhere, and no diarrhœa.

This condition continued, under a mild treatment, with little change for several days; when suddenly on the ninth day after admission, perhaps near the end of the second week from the occurrence of the first morbid feelings, the patient was seized with symptoms of severe peritonitis. The contrast between his condition on the previous day, when he seemed to be laboring under only a moderate fever, and that which he now presented on the occasion of my visit, was very striking. The abdomen was at this time much swollen, tense, and exquisitely painful upon pressure; the patient complained constantly of severe pain in this region; the decubitis was on the back with the knees drawn up; the pulse was small, thready, and extremely frequent; the skin hot; and the countenance shrunk and expressive of great anxiety. There could be no doubt of the existence of severe peritonitis; and the symptoms were found aggravated on the following day, when the tongue had become dry, and the abdomen still more distended.

Thinking that the case was one probably of perforation, Dr. W. directed the patient to be placed and maintained under the decided influence of morphia; a blister to be applied over the whole abdomen, to be followed by an emollient poultice, and one grain of the mercurial pill to be given every hour until the gums should be affected. The diet consisted of sago boiled with equal parts of milk and water, and the drink of ice-water, in small quantities. The object of the morphia was to keep the intestines as nearly immovable as possible, of the blue mass and the blister to obviate the inflammation, of the diet to support the strength already much enfeebled by the pre-existing fever. Bleeding in any shape was wholly out of the question.

This plan of treatment was steadily pursued. The amount of sulphate of morphia necessary to keep the patient in a constant state of semi-insensibility, short, however, of stupor, varied from three to five grains daily, given in divided doses, equivalent to at least from twelve to twenty grains of opium. One of its effects was to produce and sustain a steady and rather copious perspiration. The mouth became slightly affected in two or three days, after which the mercurial was gradually diminished. In three or four days some amendment was observable; and, in somewhat less than a week, the pulse had become fuller and slower, the countenance much less anxious, the abdomen softer, and the whole condition of the patient more favorable. The bowels, which had not been moved from the beginning of the peritoneal attack, were now opened spontaneously. The inflammation had obviously much subsided; but the tongue remained dry, the febrile symptoms continued, and it appeared to Dr. W. that while the patient had escaped the greatest danger of the peritonitis, he was in no little hazard from the diseased glands of Peyer, supposing the case to be, as he supposed, one of enteric or typhoid fever.

Having from much experience great confidence in the oil of turpentine in this stage of enteric fever, and believing that, if there had been perforation, the opening was now closed by adhesions, Dr. W. ventured upon this remedy in the dose of ten drops every two hours given in the form of emulsion; the morphia and blue mass being continued. In two days after commencing with the oil of turpentine, the patient exhibited evidences of decided improvement; the tongue having become moist with a disposition to clean, the tympanites sensibly diminished, and all the symptoms ameliorated. The mercurial was now omitted; but the morphia and oil were continued, though in quantities gradually diminishing with the progress of amendment. From this time convalescence fairly began; and, in the course of five or six weeks, the patient was restored to tolerable health, though with occasional symptoms of constitutional and local disturbance, such as might be expected to follow an attack so severe, protracted, and exhausting. At the present time, somewhat more than three months from his original seizure, he is still in the hospital, with a good appetite, a clean tongue, a good pulse, and greatly increased weight, but suffering with occasional abdominal pains and distension, apparently arising from accumulation of flatus, retained by the impediment offered to the regular movement of the bowels by the organized adhesions which have probably taken place.

That the peritonitis, in this case, depended on intestinal perforation cannot be positively determined; but the probabilities appeared to Dr. W. to be strongly in favor of that view; and, at all events, the case is therapeutically interesting, as showing the advantages of the opiate treatment, conjoined with mercurialization, under circumstances which did not permit a recourse to the lancet. Another point of considerable interest is, Dr. W. thought, presented in the apparently excellent effect of the oil of turpentine in obviating the symptoms of the typhoid or enteric fever, after the subsidence of the peritonial inflammation. —*Transactions of the College of Physicians Phila.*

